# Consensus on intratympanic drug delivery for Menière’s disease

**DOI:** 10.1007/s00405-022-07374-y

**Published:** 2022-04-26

**Authors:** Shuna Li, Ilmari Pyykkö, Qing Zhang, Jun Yang, Maoli Duan

**Affiliations:** 1 Department of Otorhinolaryngology-Head & Neck Surgery, Xinhua Hospital Affiliated to Shanghai Jiaotong University School of Medicine, Shanghai, China; 2 Institute of Otology, School of Medicine, Shanghai Jiaotong University, Shanghai, China; 3 Shanghai Key Laboratory of Ear and Nose Disease Transformation, Shanghai, 200092 China; 4 Hearing and Balance Research Unit, Field of Otolaryngology, School of Medicine, Faculty of Medicine and Health Technology, Tampere University, Tampere, Finland; 5 Department of Otolaryngology-Head & Neck, Audiology and Neurotology, Karolinska University Hospital, Stockholm, Sweden; 6 Division of Ear, Nose and Throat Diseases, Department of Clinical Science, Intervention and Technology, Karolinska Institute, Stockholm, Sweden

**Keywords:** Menière’s disease, Intratympanic treatment, Corticosteroid, Methylprednisolone, Dexamethasone, Aminoglycoside, Gentamicin

## Abstract

**Purpose:**

Intratympanic (IT) drug delivery receives attention due to its effectivity in treatment for Menière’s disease (MD). Due to the release of the consensuses and new evidence on IT drug delivery for MD have been published, the review with a view to supplementing the details of IT treatment of MD is indispensable.

**Methods:**

The literatures on IT injection for MD treatment over the last two decades are retrieved, International consensus (ICON) on treatment of Menière’s disease (2018), Clinical Practice Guideline (2020) and European Position statement on Diagnosis and Treatment of Meniere’s Disease (2018) are taken into account for reference, and follow advice from experts from Europe, USA and China.

**Results:**

Experts agree on the following: (1) The effectiveness of IT methylprednisolone (ITM) on vertigo control seems to be somewhat better than that of IT dexamethasone (ITD), and ITM can restore hearing in some cases. (2) Due to the ototoxicity of aminoglycosides, the application of intratympanic gentamicin (ITG) in MD patients with good hearing is conservative. However, some studies suggest that ITG with low doses has no significant effect on hearing, which needs to be further proved by clinical studies with high levels of evidence. (3) Currently, generally accepted treatment endpoint of ITG is no vertigo attack in a 12-month period or a vestibular loss in objective tests in the affected ear.

**Conclusion:**

More studies with high level of evidence are needed to evaluate the drug type, efficacy, and therapeutic endpoint of IT therapy for MD.

## Introduction

The latest diagnostic criterion for Menière’s disease (MD) was presented in 2015 by Classification Committee of the Bárány Society [1]. Diagnosis of MD is based on clinical manifestation of episodes of vertigo, sensorineural hearing loss (SNHL), fluctuating aural symptoms (hearing, tinnitus or ear fullness). Earlier criteria included endolymphatic hydrops as a pathological sign (AAO-HNS1995) but in recent definition, there is the suggestion that patients with MD have signs of endolymphatic hydrops as the endolymphatic hydrops will not explain all symptoms of MD. The aim of medical treatment for MD is to control symptoms of MD, reducing impact of vertigo, delaying progress of hearing loss, and provide good health-related quality of life. The therapy should restore the work capacity and prevent from disablement. Management of MD includes medical therapy, intratympanic (IT) therapeutic injection, ablative and non-ablative surgery, and others. When medical treatment is unable to control vertigo attacks, IT therapy and surgical procedures such as endolymphatic sac surgery (ESS) or ablative surgery is usually considered [2, 3]. Paralleled to aforementioned management, in chronic MD rehabilitation is of importance to manage vertigo, tinnitus and hearing loss physically and their consequences of well-being.

IT injections for inner ear disease were first described in 1944 [4] and had been given increasing attention in the past two decades for treatment of MD. In International Consensus (ICON) on treatment of MD, authors proposed IT corticosteroids (ITC) as second step of treatment and intratympanic gentamicin (ITG) as fourth step, namely medical destructive treatment [5]. In addition, ITC and ITG were recommended as second line and fourth line management respectively in European Position Statement on Diagnosis and Treatment of MD [6]. In Clinical Practice Guideline from USA ITC was considered as second step and ITG was recommended as 3rd step of treatment [7]. Although ITC and ITG were mentioned in the above three consensuses, drug selection, dosage, possible adverse effects of these drugs, and treatment endpoint of ITG were not discussed. Moreover, after the release of the consensuses, new evidence on IT drug delivery for MD have been published. Therefore, we decided to present this review with a view to supplementing the details of IT treatment of MD.

We retrieved literature over the last 2 decades with key words “Menière’s disease” and “intratympanic injection”. Only systematic reviews and research articles in English were included. As such, we found that two kinds of drugs, corticosteroids and aminoglycosides, were the most commonly used alone or in the combination. IT latanoprost and ganciclovir also were applied for treatment of MD. Corticosteroids included dexamethasone, methylprednisolone and aminoglycosides included gentamicin, streptomycin. Level of evidence classification (The Oxford 2011 Levels of Evidence) was used to assess literature and recommendations were given following the grading of recommendations assessment, development and evaluation (GRADE) system in this consensus.

## Intratympanic corticosteroids

ITC is recommended to be used when medication therapy is ineffective. Methylprednisolone is likely the best choice for the treatment of MD by ITC (level 2) [8]. IT methylprednisolone (ITM, 40 mg/dl) and IT dexamethasone (ITD) (4 mg/dl) injections by three times a week were of similar benefit in controlling vertigo [9]. There was a trend toward a more sustained benefit with methylprednisolone. There was no serious adverse in ITM (level 2), though in general recognition, methylprednisolone creates burning sensation when being injected into tympanum; pain score between gentamicin (40 mg/ml) and methylprednisolone (62.5 mg/ml) showed no significant difference [10].

ITD is recommended (level 2) when methylprednisolone is not available. Five consecutive daily IT injections of dexamethasone (4 mg/ml) showed better control of vertigo compared with placebo [11].

Protocol of ITC varied in concentration and frequency in the literatures (Table 1). The concentration of dexamethasone ranged from 10 mg/dl to 40 mg/ml and frequency from 3 times one week to 2 times one month apart [9, 12–15]. The concentration of methylprednisolone ranged from 40 mg/dl to 62.5 mg/ml and frequency from once a day to 2 times two weeks apart [9, 10, 16–18]. Thus, the IT delivery schedule is not settled so far, but it seems that more than one IT-injection should be delivered and is recommended. Another factor causing variability is the site of the injection in the middle ear. If the vestibular effect is the major target, the drug delivery should be targeted close to the oval window. As most of the drug delivery is targeted to anterior lower part of the tympanum, the drugs may not always reach the oval window [19, 20].

**Table 1:** Administration Protocol of ITC

Drug	Concentration	Number	Frequency	Researcher
Dexamethasone	4 mg/dl	3	Three times a week	Masoumi E et al. 2017
Dexamethasone	1 mg/ml	≈45	Every other day for 3 months	Sennaroglu L et al. 2001
Dexamethasone	4 mg/ml	3	Three consecutive daily	Albu S et al. 2016
Dexamethasone	4 mg/ml	3	Intervals of 1 every 3 days	Casani AP et al. 2012
Dexamethasone	10 mg/ml	≤ 3	Weekly	James G. et al. 2019
Methylprednisolone	40 mg/dl	3	Three times a week	Masoumi E et al. 2017
Methylprednisolone	40 mg/ml	10	Consecutive days	She W et al. 2015
Methylprednisolone	62.5 mg/ml	3	Per week for 3 weeks	Gabra N et al. 2013
Methylprednisolone	62.5 mg/ml	2	Two weeks apart	Patel M et al. 2016
Methylprednisolone	62.5 mg/ml	2	Two weeks apart	Harcourt JP et al. 2019

*ITC* intratympanic corticoid

*The authors recommend the use of ITM over ITD as the second step after medication treatment for MD (Grade B) though further researches are needed.*

### Intratympanic aminoglycosides

In consideration of potential risk of hearing loss induced by aminoglycosides, IT aminoglycosides were used to treat MD before conservative surgery, ESS, is to be planned. Gentamicin or streptomycin was used for MD treatment, while gentamicin is today preferred. When compared with ITC or with a placebo, ITG was reported to be the most efficacious medication, followed by methylprednisolone, latanoprost, dexamethasone and ganciclovir (level 2) [8].

ITG, compared with ESS, revealed better posttreatment functional levels [21]. Sennaroglu et al. [15] compared the vertigo control via ITD, ITG and ESS; ITG was more effective than ITD and followed by ESS. In this study, ITG (20 mg/ml, 3 times a day for 1 week) showed significant ototoxicity taking no account of hearing level consistency before treatment and vertigo attacks was evaluated according to AAO-HNS 1985 criterion [22] at the end of 18 months [15]. In a retrospective study, ESS showed same vertigo control as ITG but a lower incidence of audiovestibular complications [23]. A Meta-analysis showed that ITG is superior to ITC in reducing the number of vertigo attacks in the treatment of MD significantly; there was no clear difference between ITG and ITC on hearing improvement and on hearing loss [24].

Recently, some studies indicated that low-dose gentamicin injection in middle ear did not lead to hearing loss but longitudinal evaluation of the efficacy was missing. Patel et al. [10] reported that hearing levels did not significantly change from baseline after two gentamicin (40 mg/ml) injections were given 2 weeks apart. After dexamethasone (10 mg/ml) administration weekly up to three injections and gentamicin (26.7 mg/ml) administration every 2 weeks up to three injections respectively till vertigo control was achieved, change in pure tone audiometry from baseline was not different between treatment groups [12]. After single dose of gentamicin, about half patients developed spontaneous nystagmus or post-headshaking nystagmus. ITG causes partial vestibular lesions that involve preservation of spontaneous discharge and /or rotational sensitivity of afferents [25]. Previous studies suggested that ITG should be immediately terminated if there are obvious ototoxic adverse effects (hearing loss and vestibular reaction) [26]. In one report, hearing loss was observed in about 10% patients after 4 doses of ITG but less than 5% after 2 doses [27]. Note should be given that mutation A1555G could cause significant hearing loss even with one ITG injection. [28].

There were also a few articles revealed that IT injection of a mixture of gentamicin and dexamethasone (ITG + D) in intractable Meniere’s disease cases was more effective than dexamethasone or gentamicin alone for vertigo control [29, 30]. Two years after treatment there was better control of vertigo in mixture group (ITG + D) than in ITD alone (Table 2).

**Table 2:** Dose, endpoint and effect of ITG

Concentration(mg/ml)	Number	Frequency	Endpoint of treatment	Effect		Researcher
				Vertigo	Hearing loss	
26.7	1–3	Every week	Vertigo was controlled	Vertigo control rate is 100%	No	Carey JP et al. 2002
26.7	3–4	Every week	Vertigo was controlled	87% reduction	No	Paradis J et al. 2013
26.7	≤ 3	2 weeks apart	No vertigo attacks and/or objective testing revealed a vestibular loss	Vertigo control rate is 0% in 2 years	No	Naples JG et at. 2019
30	1-several	/	Significant hearing deterioration, vertigo attacks stopped, or less than one vertigo attack per month	Vertigo control rate is 66.8%	No	Gibson AW et al. 2019
40	2	2 weeks apart	No vertigo attacks	87% Reduction	No	Patel M et al. 2016
40	1–5	2 weeks apart	Vertigo was controlled	4.4 Vertigo attacks/month decreased to 0.52	No	Scarpa A et al. 2019
40	1–2	5 weeks apart	vHIT gain diminished to a value below the lower limit of normal/a 10 dB decrease of PTA	Vertigo control rate is 70%	/	Martin-Sanz E et al. 2019

*ITG* intratympanic gentamicin; *vHIT* video-head impulse test; *PTA* pure tone audiometry

### Endpoint of ITG treatment

In the common view, a maximum of 3–5 ITG injections could be performed in each patient. Treatment was stopped when the patient had no episodes of vertigo in a 3 month period [31] or objective testing (videonystagmography, rotatory chair) revealed a vestibular loss in the affected ear [12].Vestibular ocular reflex (VOR) changes after ITG in video-head impulse test (vHIT) could foresee short-term control of vertigo attacks. If the difference value of gain asymmetry between the symptomatic and asymptomatic ear in the horizontal semicircular canal was greater than 7% and the amount of vestibular function reduction in the symptomatic ear, evaluated with the VOR gain, was greater than 17.8%, the second ITG injection could be avoided [32]. Another study found that horizontal VOR gain can predict a higher vertigo-free period if it decreased by ITG after treatment in 1 month more than 33% [33]. If VOR gain difference of horizontal canal is relatively low after initial ITG, patient might have poor vertigo control and be required another ITG [34]. When vestibular-evoked myogenic potentials (VEMPs) were evaluated for any significant prediction, posttreatment general dizziness status VAS scores in MD patients were found highest in the group with absent VEMPs indicating better outcome. Hence, VEMPs absent at post-treatment second week was significant predictor of post-treatment 6-month dizziness status and vertigo control in unilateral MD patients [35]. More research is needed on the application of vestibular function tests to predict therapeutic endpoints of ITG.

*ITG administration two times 2 weeks apart is recommended to control symptoms of MD when ITC is ineffective. The authors also recommend the use of ITG when patients with hearing loss instead of ESS or have contraindications for surgical treatment (Grade B). However, the concentration of gentamicin and frequency of ITG need further substantial evidence.*

### Intratympanic other drugs

Due to small sample size, effectiveness of latanoprost needs to be interpreted cautiously even though a placebo-controlled double-blind study design was adopted [8] (level 2). There was no evidence to support the effectiveness of IT antivirals and latanoprost [36]. A cohort study indicated improvement in vertigo, aural fullness and less vertigo after repetitive IT administration of lidocaine [37] (level 1).

### Summarization

**Fig. 1:**
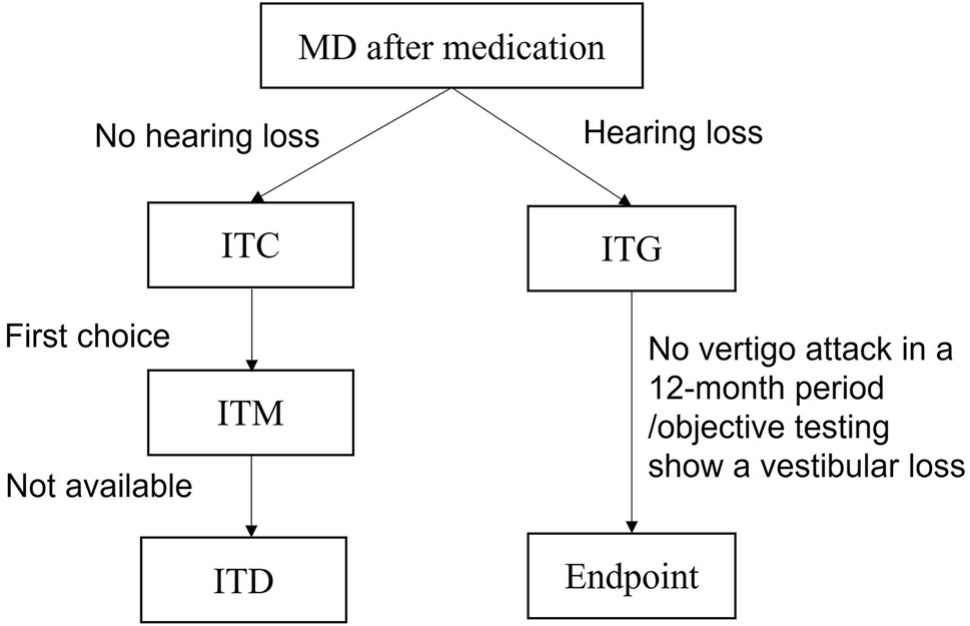
A Algorithm proposed of intratympanic drug delivery for MD. *MD* Menière’s disease, *ITC* Intratympanic corticosteroid, *ITM* intratympanic methylprednisolone, *ITD* intratympanic dexamethasone, *ITG* intratympanic gentamicin

In conclusion, we proposed the algorithm for IT drug delivery for MD (Fig. 1) and put forward following points: (1) the effectiveness of ITM on vertigo control seems to be somewhat better than that of ITD, and ITM can restore hearing in some cases. (2) Due to the ototoxicity of aminoglycosides, the application of ITG in MD patients with good hearing is conservative. However, some studies suggest that ITG with low doses has no significant effect on hearing, which needs to be further proved by clinical studies with high levels of evidence. (3) Currently, generally accepted treatment endpoint of ITG is no vertigo attack in a 12-month period or a vestibular loss in objective tests in the affected ear. The significance of VEMP and vHIT for the endpoint of ITG treatment needs further study.
